# Voltammetric Measurement of Antioxidant Activity by Prevention of Cu(II)-Induced Oxidative Damage on DNA Bases Using a Modified Electrode

**DOI:** 10.1021/acsomega.2c08055

**Published:** 2023-01-23

**Authors:** Ayşe Nur Önem, Kevser Sözgen Başkan, Reşat Apak

**Affiliations:** † Department of Chemistry, Faculty of Engineering, Istanbul University-Cerrahpaşa, Avcilar, Istanbul 34320, Turkey; ‡ Turkish Academy of Sciences (TUBA), Çankaya, Ankara 06690, Turkey

## Abstract

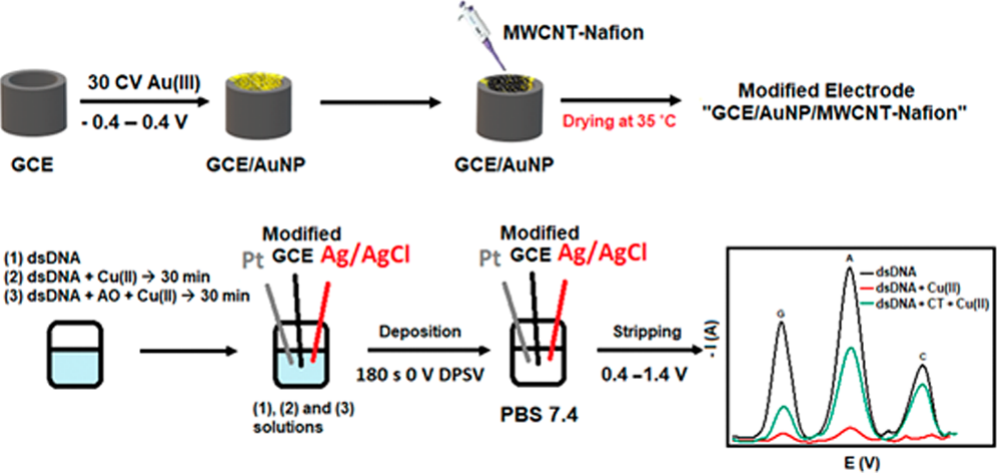

The protective effect of antioxidants using electrochemical techniques can be evaluated by examining the oxidative changes in deoxyribonucleic acid (DNA) nucleobases. In this study, a gold nanoparticle (AuNP)-decorated and multiwalled carbon nanotube (MWCNT)-Nafion-modified glassy carbon electrode (GCE/AuNP/MWCNT-Nafion) was developed to evaluate the preventive ability of antioxidants on oxidative DNA damage. A modified working electrode was prepared and characterized by cyclic voltammetry, electrochemical impedance spectroscopy, and scanning electron microscopy. The developed electrochemical method relies on two phenomena: (i) reactive species (RS) produced by dissolved oxygen in the presence of copper(II) partially damage the DNA immobilized on the electrode surface and (ii) antioxidant compounds prevent this damage by scavenging the formed RS. Changes in guanine, adenine, and cytosine oxidation signals resulting from DNA damage were measured using differential pulse stripping voltammetry before/after the interaction of dsDNA with Cu(II) while antioxidants were absent or present. The DNA protective ability of antioxidants was assessed for a number of antioxidant compounds (i.e., ascorbic acid, gallic acid, epicatechin, catechin, epicatechin gallate, glutathione, chlorogenic acid, *N*-acetyl cysteine, rosmarinic acid, quercetin, and rutin). Quercetin was found to show the highest antioxidant effect, and its limit of detection was determined as 1 μM. The manufactured biosensor was put in an application for the determination of antioxidant activity of herbal teas.

## 1 Introduction

Deoxyribonucleic acid (DNA) is a complex biomacromolecule that controls vital hereditary properties of living organisms. Genes are essential DNA segments secondarily associated with protein coding, which serve as pivotal structures for the cellular system.^1−3^ The two strands of the DNA molecule, on which simpler units known as nucleotides are located, constitute a double helix. Each nucleotide contains a nitrogenous cyclic nucleobase: adenine (A), guanine (G), cytosine (C), or thymine (T) attached to a backbone of monosaccharide sugar and phosphate organized in a framework.^4^ The integrity of the human genome is prone to inner/outer effects harming DNA. Reactive oxygen species (ROS), continuously and spontaneously generated during metabolism (i.e., via respiration, since molecular oxygen can only accept electrons in a stepwise manner due to the spin restriction in its π*-antibonding orbitals), are important agents that may cause DNA damage. ROS comprises various species, e.g., superoxide anion radical (O_2_^•–^), hydrogen peroxide (H_2_O_2_), hydroxyl radical (^•^OH), and singlet oxygen (^1^O_2_) that emerge as a result of stepwise electron uptake of O_2_. If not counterbalanced by natural or dietary antioxidative defenses of the organism, reactive oxygen/nitrogen species can bring about oxidative stress and consequently a variety of critical illnesses, e.g., cardiovascular, inflammatory, and neurological disorders, gene mutations, and cancer progression.^5−7^ Most of the hydroxyl radicals in living organisms originate from transition metal (M) ion-induced disintegration of hydrogen peroxide. In the presence of transition metal ions in their lower oxidation states, e.g., ferrous, cuprous, or cobaltous ions, H_2_O_2_ is converted to ^•^OH and OH– by one-electron uptake, commonly known as Fenton-type redox reactions.^8^ Chumakov et al.^9^ reported the ability of certain metal ions to generate ^•^OH when they react with electrogenerated hydrogen peroxide (formed by cathodic reduction of dissolved molecular oxygen) using voltammetry and determined its efficacy order as Cr^3+^ > Ce^3+^ > Cu^+^ > Co^2+^ > Fe^2+^ > Ni^2+^ > La^2+^.

Most living organisms are equipped with multilevel endogenous defensive strategies that repair damaged DNA through specific enzymes involved in base excision repairs, such as superoxide dismutase, catalase, peroxidase, and myeloperoxidase. In this regard, exogenous antioxidant (AO) compounds, e.g., vitamins (A, E, C, and β-carotene), phenolics, minerals (Se and Zn), or proteins (albumin, catalase, transferrin, and ceruloplasmin) may provide extra protection.^8,10^ According to the findings in recent years, AOs have an anti-ROS protective role in biomacromolecules such as proteins, DNA, and others. Cellular-level protection by AOs is closely associated with maintaining DNA integrity. In recent times, numerous bioelectrochemical sensors targeting DNA have been designed for evaluating either AO capacity or activity.^7,8,10−13^ It is reasonable to use DNA biosensors for evaluating antioxidative efficiency because the measurement principle of these biosensors simulates the actual AO action in biosystems. Also, monitoring AO activity (AOA) by electrochemical biosensors based on the redox principle has many advantages over traditional chemical methods. This technique neither requires chemical reagents or complex solvents nor special handling of samples. It provides comprehensive and repeatable information about electrokinetic processes and enables the rapid implementation of tests.

In recent years, DNA-based electrochemical sensors have been developed to evaluate AOA. Voltammetry, in the form of cyclic (CV), square wave (SWV), and differential pulse (DPV), is the most widely used electrochemical technique for these studies.^6,11,13^ There are many studies on the electrochemical analysis of DNA or its nucleobases using electrodes such as carbon nanotube (CNT)-modified screen-printed electrodes (SPEs),^14^ glassy carbon electrodes (GCEs),^15^ and pencil graphite electrodes (PGEs).^16−19^ For instance, in the study by Mello et al., a DNA layer was immobilized on an SPE as a biosensor, and a Fenton-type reaction was used to produce damage by generating ^•^OH radicals. In this study, the changes in the G oxidation peak as a result of the interaction of immobilized DNA with radical species in the presence and absence of AOs were measured by the SWV method. Aqueous plant extracts were used as AO sources, and their AO effects were determined.^20^ In another study, a biosensor prepared by electro-immobilization of a purine base (G or A) on the GCE surface was used to evaluate the total AO capacity of beverages. In this study, the interaction between the immobilized purine base and the free radicals in the absence and presence of AOs was evaluated by means of changes in the G and A anodic peaks obtained by SWV.^10^ Another biosensor prepared by modifying a carbon film electrode with dsDNA and chitosan layer-by-layer coverage could detect O_2_^•–^ and ^•^OH oxidative damage on DNA using CV, SWV, and electrochemical impedance spectroscopy (EIS) methods. The developed method was used to determine the AO effects of commercial apple and orange juices.^21^ In the study by Uzunboy et al.,^22^ the DNA damage was electrochemically investigated using PGEs as a DNA sensor platform in combination with DPV; the interaction of the radical species with DNA in the absence/presence of AOs was detected according to the changes in the G oxidation signal. In the electrochemical method developed to measure the AO potential and protective effect of *Acanthophora* red macroalgae collected from the Persian Gulf coast of Bushehr, Iran, a biosensor was designed to prevent DNA damage caused by the Fenton reaction. This biosensor was prepared by immobilizing the gene probe of human interleukin-2 (IL-2) on the surface of a gold nanoparticle (AuNP)-modified carbon SPE; then, the protective effect of the algae extract was investigated by EIS.^7^

In this study, a AuNP-decorated multiwalled carbon nanotube (MWCNT)-Nafion-modified GCE (GCE/AuNP/MWCNT-Nafion) was developed to evaluate the AO ability in preventing oxidative DNA damage. The prepared modified working electrode was characterized by CV, EIS, and scanning electron microscopy (SEM) techniques. The developed electrochemical method is based on the fact that the ROS produced by dissolved oxygen in the presence of copper(II) partially damage the DNA immobilized on the electrode surface and that AO compounds prevent this damage by scavenging the formed ROS. Copper(II) was selected as an inducer of DNA oxidation because it can bind to DNA and produce site-specific damage that may give rise to other ROS. Changes in the oxidation signals of G, A, and C nucleobases resulting from DNA damage were measured using differential pulse stripping voltammetry (DPSV) before/after the interaction of dsDNA with Cu(II) either in the absence or the presence of certain AO compounds, i.e.*,* ascorbic acid, gallic acid, catechin, epicatechin, epicatechin gallate, chlorogenic acid, rosmarinic acid, quercetin, rutin, glutathione, and *N*-acetyl cysteine. In addition, the obtained analytical findings were compared with those of the high-performance liquid chromatography (HPLC) method. Finally, the effects of some interferences that can be found in human serum on the developed electroanalytical method were investigated.

## 2 Methods

### 2.1 Chemicals

The analytical reagent grade chemicals and their sources are listed as follows: hydrochloric acid (HCl), nitric acid (HNO_3_), sodium hydroxide (NaOH), copper(II) sulfate pentahydrate (CuSO_4_·5H_2_O), potassium hexacyanoferrate(III) (K_3_[Fe(CN)_6_]), potassium hexacyanoferrate(II) (K_4_[Fe(CN)_6_]), and potassium chloride (KCl) from Merck Chemicals (Darmstadt, Germany); gallic acid (GA), chlorogenic acid (CA), (−)epicatechin (EC), quercetin (QR), methanol (MeOH), absolute ethanol (EtOH), sodium acetate (CH_3_COONa), *N*-acetyl cysteine (NAC), l-glutathione reduced (GSH), and disodium monohydrogen phosphate (Na_2_HPO_4_) from Sigma-Aldrich Chemicals (Steinheim, Germany); (−)-catechin (CT) and sodium chloride (NaCl) from Fluka (Buchs, Switzerland); double-stranded DNA (dsDNA, fish sperm) from Serva Electrophoresis GmbH; guanine (G), HAuCl_4_·3H_2_O (99.999%), carbon nanotube, multiwalled (MWCNT, carbon >95% O.D. × L 6–9 × 5 μm), dimethylformamide (DMF), (−)epicatechin gallate (ECG), rosmarinic acid (RA), and Nafion (5 wt % in lower aliphatic alcohols) from Aldrich Chemicals Co. (Steinheim, Germany); ascorbic acid (AA), rutin hydrate (RT), cytosine (C), adenine (A), fetal bovine serum (FBS), bovine serum albumin (BSA), dopamine (DA), glucose, and uric acid (UA) from Sigma (St. Louis, MO, USA); acetic acid (CH_3_COOH), phosphoric acid (H_3_PO_4_), sodium dihydrogen phosphate (NaH_2_PO_4_), and sulfuric acid (H_2_SO_4_) from Riedel-De-Haën (Germany).

### 2.2 Apparatus

The electrochemical investigations (CV, EIS, and DPSV) were carried out with the use of a potentiostat model Reference 600 (Potentiostat/Galvanostat/ZRA, Gamry). A three-electrode system, employing a GCE (Gamry, 3 mm disk, 7 mm OD) as the working electrode, platinum wire as the counter electrode, and Ag/AgCl (3.0 M KCl) as the reference electrode, was used. The Varian Cary 100 Bio model UV–vis region spectrophotometer and a pair of matching quartz cuvettes with 10 mm light path were used for spectrophotometric measurements. An HPLC system (Waters Breeze 2 model, Milford, MA, USA) equipped with a 1525 binary pump, a column oven with a thermostat, a 2998 photo-diode array (PDA) detector (Chelmsford, MA, USA), and a Hamilton 25 μL syringe (Reno, NV, USA) was used for chromatographic measurements. Data collection was performed using Empower PRO (Waters Associates, Milford, MA). SEM with field emission (FEI Quanta 450 FEG) imaging device for surface characterization of electrodes and a microwave extraction system (Milestone ETHOS One) for preparing extracts of plant samples were used. The solution pH values were adjusted using a Hanna HI 221 pH meter.

### 2.3 Preparation of Solutions

A stock dsDNA solution (1000 mg/L) was prepared in a pH 4.8 acetate buffer solution at a 50 mM concentration, in which 20 mM NaCl was dissolved to adjust the ionic strength. Carboxylation of the MWCNT was performed by sonicating the MWCNT (0.05 g) in 60 mL of 2.2 M HNO_3_ for 20 h (at room temperature). Then, it was washed with distilled water until neutral and oven-dried at 37 °C.^23^ An MWCNT solution was prepared with a DMF solvent at a 2 mg/mL concentration using the dried MWCNT and was kept in an ultrasonic water bath for 30 min. A Nafion solution containing 0.01% (v/v) was added to the prepared MWCNT (10 mL) and used in the modification (MWCNT-Nafion). Before each modification, it was kept in an ultrasonic water bath for 5 min. AO solutions (10 mM) were prepared with bidistilled water. Quercetin and rutin standards were prepared to contain 10% (v/v) EtOH. A HAuCl_4_ solution containing 0.04% (w/v) gold(III) chloride, used for coating the AuNPs on the GCE surface, was prepared in distilled water.

Extraction of plant samples was carried out by the microwave extraction system in Teflon (polytetrafluoroethylene, PTFE) containers, using fiber optic temperature control and a closed oven system.^24^ The plant samples (green tea, apple tea, echinacea, and linden) were extracted with the use of a previously published method that is slightly modified: A dried and ground plant sample (1.0 g) in 15 mL bidistilled water was placed in the inner vessel, into which a magnetic rod (for stirring) was mounted. The suspensions were irradiated with microwaves in the following manner: heating to the desired temperature (80 °C) took 3 min, conditioned at 80 °C for another 3 min, and finally cooled for 5 min. Plant extracts obtained were filtered through a 0.45 μm PTFE syringe filter and stored at +4 °C until analysis.

### 2.4 Glassy Carbon Electrode Pretreatment

A bare GCE was pretreated carefully with 0.05 μm alumina slurry on a polishing cloth and then washed with distilled water. Following each treatment, the electrode was consecutively ultrasonicated for 10 min in distilled H_2_O and pure EtOH to remove the remaining Al_2_O_3_ particles left on the surface. The clear GCEs were initially electroactivated in the buffer medium using 300 s, 1.75 V chronoamperometry, and then CV (0.3–1.25 V, 5 cycles) was applied for their stabilization.^25^

### 2.5 Preparation of the Modified Working Electrode (GCE/AuNP/MWCNT-Nafion)

**Scheme 1:**
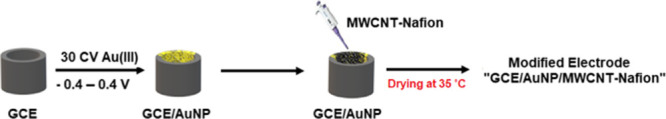
Schematic Diagram of GCE/AuNP/MWCNT-Nafion Biosensor Preparation

The GCE surface was coated with AuNPs by electrodeposition using 0.04% (w/v) HAuCl_4_ (2.5 mL) + 0.1 mol/L H_2_SO_4_ (2.5 mL) solutions.^26^ For this purpose, 30 cycles of the coating were performed with the CV technique at a scan rate of 50 mV/s in the range (−0.4 to +0.4 V). The MWCNT-Nafion solution was dripped onto the surface of the GC electrode coated with AuNPs (optimal volume: 3.5 μL) and left to dry in an oven at 35 °C to make the modified working electrode (GCE/AuNP/MWCNT-Nafion) ready for analysis (Scheme 1). Modified electrodes were characterized in 0.1 M KCl containing 5 mM K_3_[Fe(CN)_6_]/K_4_[Fe(CN)_6_] (1:1) using CV at a potential range of −0.2 to +0.6 V at a scan rate of 50 mV/s and using EIS with a frequency interval of 10^6^–10^–1^ Hz at an amplitude of 5 mV. Finally, the topographical characterization of the surface was made using SEM to observe the alteration of the electrode surface.

### 2.6 Assay Procedures

All experiments were carried out at room temperature. A new modified electrode was used before each electrochemical measurement.

#### 2.6.1 UV–Vis Spectrophotometric Measurements

In order to examine the formation of DNA damage in the presence of dissolved oxygen with Cu(II), the UV–vis spectra of the dsDNA solutions (in pH 7.4 PBS buffer) were taken in the range of 200–500 nm before and after damage. Their absorbances at 260 nm, i.e., the characteristic wavelength of DNA, were compared.

#### 2.6.2 DPSV Measurements

Each test was repeated six times, and average values were presented in the graphics with error bars.

##### 2.6.2.1 Determination of Undamaged dsDNA

The modified electrode (GCE/AuNP/MWCNT-Nafion) was immersed in the cell containing 1 mL of 1000 mg/L dsDNA and 3 mL of 75 mM pH 7.4 PBS solutions. DNA was deposited on the electrode surface for 180 s at 0 V potential.^27^ Then, the electrodes were washed with bidistilled water and immersed in a clean cell containing 4 mL of the 75 mM pH 7.4 PBS solution, and stripping of the dsDNA immobilized on the electrode surface was performed (scan range: 0.4–1.4 V).

##### 2.6.2.2 Determination of Damaged dsDNA (Cu(II)-Catalyzed Damage Method)

For the oxidative damage of DNA, a Cu(II)-catalyzed damage method available in the literature was applied with modification.^28^ Accordingly, 1 mL of 1000 mg/L dsDNA, 2 mL of 75 mM pH 7.4 PBS, and 1 mL of 6 mM Cu(II) solutions were added to an electrochemical cell. It was then incubated for 30 min in a laboratory environment, leaving it open to air O_2_. After incubation, the modified electrode (GCE/AuNP/MWCNT-Nafion) was immersed in this solution, and undamaged DNA was deposited on the electrode surface at 0 V potential for 180 s. Then, the electrodes were washed with bidistilled water and immersed in a clean cell containing 4 mL of the 75 mM pH 7.4 PBS solution, and the undamaged DNA was stripped from the surface (scan range: 0.4–1.4 V).

##### 2.6.2.3 Determination of AO Ability in Preventing Oxidative DNA Damage

**Scheme 2:**
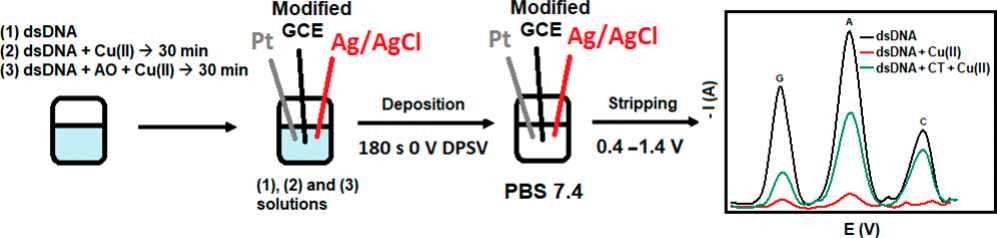
Schematic Diagram of the Developed Electrochemical Method for the Determination of AO Ability in Preventing DNA Damage

The effect in preventing oxidative DNA damage of certain AOs (QR, RT, AA, GA, CA, CT, EC, ECG, RA, GSH, and NAC) was investigated. For this purpose, 1 mL of 1000 mg/L dsDNA, 0.5 mL of (*x*) mM AO, 1.5 mL of 75 mM pH 7.4 PBS, and 1 mL of 6 mM Cu(II) solutions were added into an electrochemical cell and incubated for 30 min (in the presence of dissolved O_2_). After incubation, the modified electrode (GCE/AuNP/MWCNT-Nafion) was immersed in the prepared solution, and undamaged DNA (protected by AO) was deposited on the electrode surface at 0 V potential for 180 s. The electrodes were then washed with bidistilled water and immersed in a clean cell containing 4 mL of the 75 mM pH 7.4 PBS solution, and the undamaged DNA was stripped from the surface (scan range: 0.4–1.4 V) (Scheme 2). The linear operating ranges in which the tested AOs showed a protective effect were determined by using AO solutions prepared at different concentrations (*x* = 0.005–1.5 mM). Using the same procedure, the AO abilities of plant samples in preventing oxidative DNA damage (0.01, 0.025, and 0.05 mL) were investigated.

#### 2.6.3 HPLC-PDA Measurements

The results found with the electrochemical method for the determination of AO ability in preventing oxidative DNA damage were compared to those of a published HPLC method using a PDA detector.^29^ Solutions containing 0.005–0.05 mM CT and 0.01–0.05 mL of green tea extract were separately injected into the HPLC system, and analyses were carried out simultaneously with both electrochemical and chromatographic methods. The HPLC-PDA analysis was performed with a Symmetry C_18_ (i.d. 5 μm, 250 × 4.6 mm) column (Waters, MA, USA) at a flow rate of 0.8 mL/min (isocratic elution). The mobile phase was made up of 0.01 M PBS (pH 7.0) and methanol (85:15, v/v). Before injection, the sample solutions were properly filtered through 0.45 μm PTFE syringe filters. The column temperature was selected as 25 °C, and the detection wavelength was 254 nm.

#### 2.6.4 Interference Effects on the DPSV Method

The interference effects of some compounds that are likely to be found in biological sample matrices were investigated in DNA analysis with the developed electroanalytical method. In this regard, DA, UA,^30^ proteins (represented by BSA), and glucose were investigated as possible interferents of biological significance. In addition, the effect of FBS (protein-rich, synthetic serum containing hormones and enzymes) matrix was also investigated. In the analysis of a 250 mg/L dsDNA solution with the developed DPSV method, 41 mg/L BSA, 44 mg/L UA, 790 mg/L glucose, 8 mg/L DA, and 1:1 diluted FBS (0.01–0.05 mL) solutions were used. The effects of each were examined one by one in three different applications, including before the interaction of DNA with Cu(II), after 30 min of interaction, and in the presence of 0.05 mM GA as AO. The results were evaluated with their percentage errors.

### 2.7 Statistical Analysis

Descriptive statistical analyses were performed using Excel software (Microsoft Office 2013) for calculating the means and the standard error of the mean. Results were expressed as the mean ± standard deviation (SD). Statistical comparison of the proposed method was made by means of Student’s *t*- and *F*-tests for evaluating accuracy and precision, respectively.

## 3 Results and Discussion

### 3.1 Electrochemical Behavior of DNA on the Modified Working Electrode

Since the current values obtained with the bare GCE were very low, it was decided to modify the electrode so as to enhance the signals, and the electrodes were prepared by coating the GCE surface with different materials. CNTs are one of the most powerful nanomaterials used to improve surface properties.^31,32^ CNTs can be used for DNA determination in combination with AuNPs, by exploiting the high surface area together with the good conductivity of CNTs.^33^ In addition, it has been reported in the literature that CNTs are functionalized substrates to build efficient electrochemical DNA sensors.^34^ The surface-confined MWCNT facilitates the adsorptive deposition of nucleobases and causes a significant enhancement in the oxidation signal.^19^ Nafion, on the other hand, reduces the aggregation and recrystallization of nanoparticles, causing them to be fixed on the electrode surface, thus increasing the stability of the sensor.^35^ Thus, AuNPs and MWCNT-Nafion were combined to modify the GCE, and the new GCE/AuNP/MWCNT-Nafion electrode was produced to determine both the oxidative DNA damage and its prevention by AOs.

**Figure 1:**
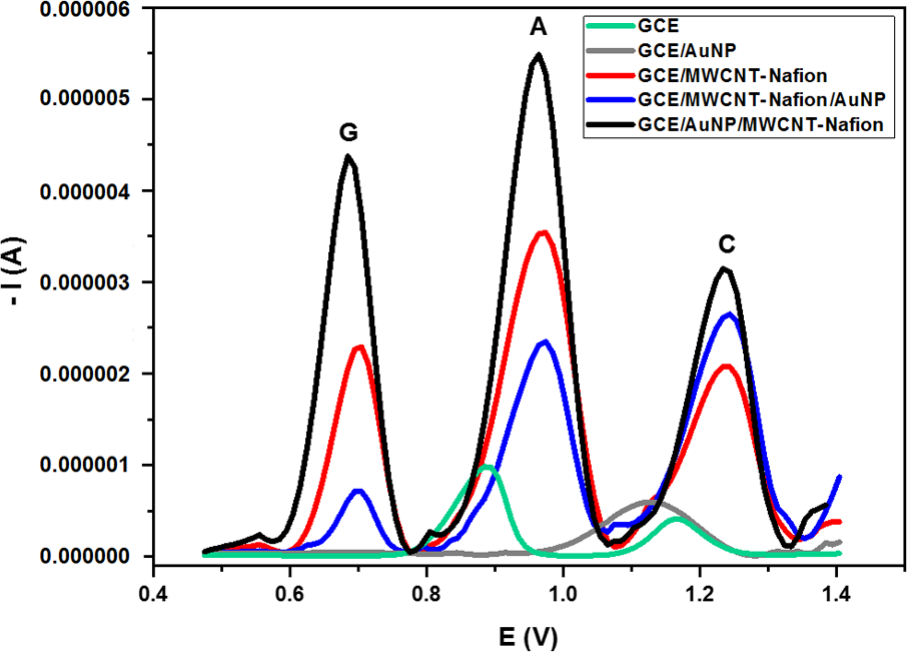
Comparison of different modes of modification of the bare electrode with AuNP, MWCNT, and Nafion in the presence of 250 mg/L dsDNA [scan range: (+) 0.4 to (+) 1.4 V; step size: 5 mV; pulse size: 25 mV; sample period: 0.5 s; pulse time: 0.1 s; deposition time: 180 s; deposition potential: 0 V; and 50 mM pH 7.4 PBS].

Some preliminary work was performed to develop the biosensor electrode capable of measuring both the DNA damage and its preventive AO ability. First, the alterations in the redox properties of DNA (i.e., nucleobase oxidation) were followed to understand the DNA electrochemistry on the modified electrode. Figure 1 shows the oxidation signals of DNA bases obtained using the DPSV method with five different electrodes, namely bare GCE, GCE/AuNP, GCE/MWCNT-Nafion, GCE/MWCNT-Nafion/AuNP, and GCE/AuNP/MWCNT-Nafion. The MWCNT-Nafion-modified electrode provided enhanced oxidation signals compared to the bare GCE due to the increased amount of immobilized DNA as a result of MWCNT-Nafion interaction with DNA. On the bare electrode, on the other hand, the base signals overlapped with each other and were of low sensitivity. The results obtained with GCE/AuNP/MWCNT-Nafion appeared to offer a clearly improved signal when compared to those of other electrodes (Figure 1). Although the three bases (G, A, and C) of DNA could be detected in almost all modifications, the oxidation peak of the thymine (T) base could not be determined. Since the oxidation of pyrimidines occurs at high positive potentials, they were more difficult to detect. In addition, the oxidation currents observed for pyrimidine bases (T and C) at the same concentrations were much lower than those observed for purine bases (G and A).^36^ For these reasons, the thymine nucleobase may not have been observed. Based on this, the study was carried out by processing the signals of only three bases (G, A, and C). This result confirms the literature studies reporting that the oxidation of all DNA bases on GCEs can be detected voltammetrically, but especially purine bases are more easily detected than pyrimidine bases.^37^ In addition, Oliveira-Brett et al.^36^ stated that the identification of oxidative changes in DNA by electrochemical means is based on the detection of oxidation peaks, especially of purine bases.

As a result, it was observed that three DNA bases (G, A, and C) were easily separated in the presence of the GCE/AuNP/MWCNT-Nafion-modified electrode and could be measured at high oxidation current values, clearly demonstrating the superior performance of this electrode. In these measurements made against the Ag/AgCl reference electrode, the oxidation peaks of the G, A, and C bases were found at 0.68, 0.96, and 1.24 V, respectively. Here, the G peak was seen at a low voltage, indicating electrocatalysis with CNTs.^38^ These determined potential values were in agreement with the values found by Wang et al. with the modified GCE (i.e., 0.65, 0.93, and 1.27 V),^1^ justifying the continuation of further measurements with the GCE/AuNP/MWCNT-Nafion electrode.

### 3.2 Optimization of the Modified Working Electrode

**Figure 2:**
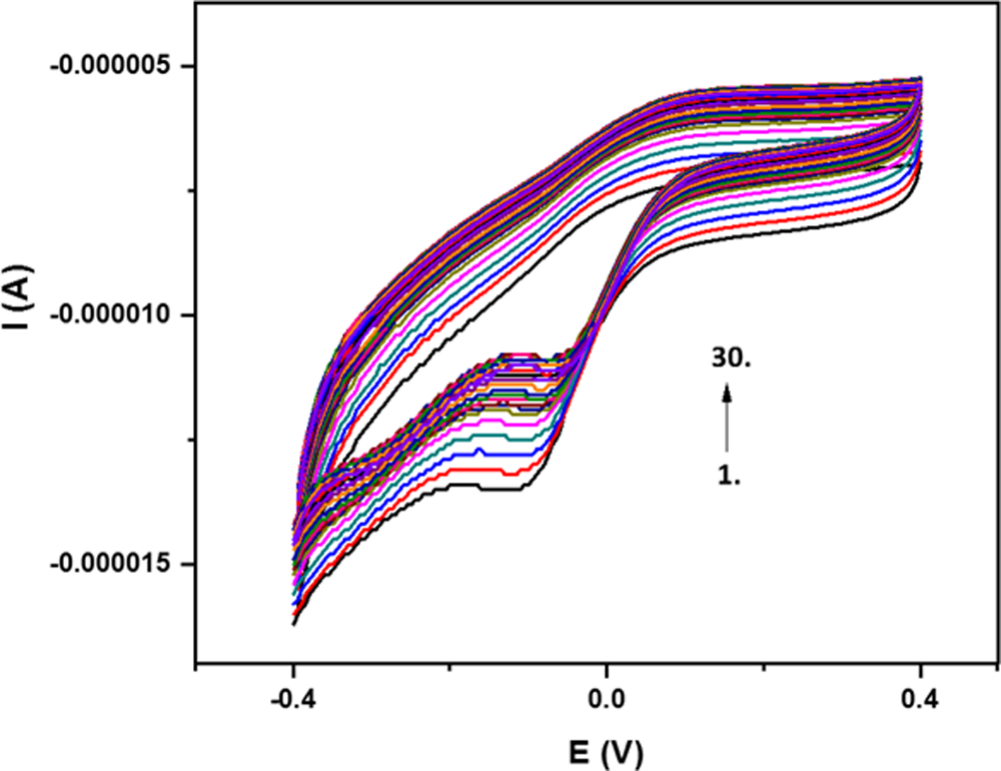
Electrodeposition coating of AuNPs on the bare electrode surface (GCE) by the CV technique (2.5 mL of 0.04% (w/v) HAuCl_4_ + 2.5 mL of 0.1 M H_2_SO_4_; scanning rate: 50 mV/s; cycle number: 30; and scanning range: −0.4 to 0.4 V).

The amounts of AuNPs and MWCNT-Nafion were optimized by DPSV to obtain the highest oxidation peak currents of DNA bases. The amount of AuNPs deposited on the surface and their effect on the measured signals were determined by optimizing the number of cycles. The peak current intensities obtained with the electrodes coated with AuNPs at different cycle numbers (10, 20, 30, 40, 50, and 60) were compared to observe 30 cycles as the optimal number (Figure 2). In the first cycle of AuNP deposition on the bare electrode surface, a reduction peak around −0.1 V was observed, the current of which corresponded to the amount of Au^3+^ ions in the medium. With increasing cycles, Au^3+^ ions in solution were reduced to Au^0^ and formed AuNPs on the surface. However, a high number of cycles (>30) caused a decrease in the oxidation currents due to the increase in surface thickness. In the second stage of modification, different amounts of the MWCNT solution containing 0.01% (v/v) Nafion were dropped onto the surface so as to set the most appropriate volume of MWCNT-Nafion to be 3.5 μL.

### 3.3 Characterization of the Modified Working Electrode

**Figure 3:**
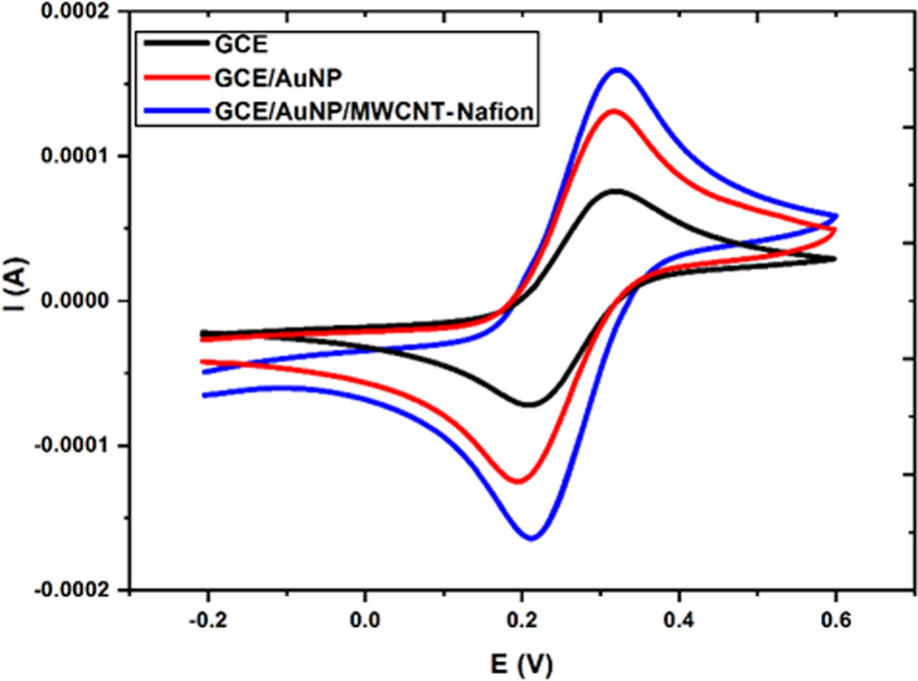
Cyclic voltammetric behavior of GC electrodes in 0.1 M KCl containing 5 mM Fe(CN)_6_^3−/4−^ (scan rate: 50 mV/s).

Various cyclic voltammetric scans were performed to examine the electrochemical behavior of the electrodes modified with AuNP and MWCNT-Nafion. In this way, the reversibility of bare GCE, AuNP, and AuNP/MWCNT-Nafion-modified electrodes was tested by CV in a 0.1 M KCl solution containing a 5 mM Fe(CN)_6_^3–/4–^ redox probe. CV measurements were performed at a scanning rate of 50 mV/s against the Ag/AgCl reference electrode between −0.2 and +0.6 V (Figure 3). The peak potential for the oxidation of the redox probe was at approximately +0.36 V and for the reduction around +0.19 V (Δ*E*_p_ = 0.17 V). For GCE/AuNP/MWCNT-Nafion, these values were found to be approximately +0.31 and +0.21 V (Δ*E*_p_ = 0.10 V), respectively. Thus, a favorable combination of these materials caused an apparent improvement in electrode reversibility.^39^ It was seen that the oxidation and reduction peak currents of this redox couple were higher with the modified electrode, the concerned anodic and cathodic peak current intensities being 197 and −204 μA, respectively.

The electrochemical response of the bare GCE compared to that of the nanomaterial-modified electrode (Figure 3) was quite low, clarifying the favorable role of modification materials to improve the redox currents of the probe. This voltammogram also revealed that the electrode material did not show any other electroactivity in this range.

Active surface areas were calculated using the “Randles–Sevcik” equation^40^ to evaluate the conductivities of bare, AuNP, and AuNP/MWCNT-Nafion-modified electrodes. The active surface area of these electrodes was calculated to be 0.0023, 0.0051, and 0.0075 cm^2^, respectively. These results show that the surface of the GCE/AuNP/MWCNT-Nafion electrode has a higher potential for catalytic activity and sensitive determinations than its counterparts due to its increased active surface area.

**Figure 4:**
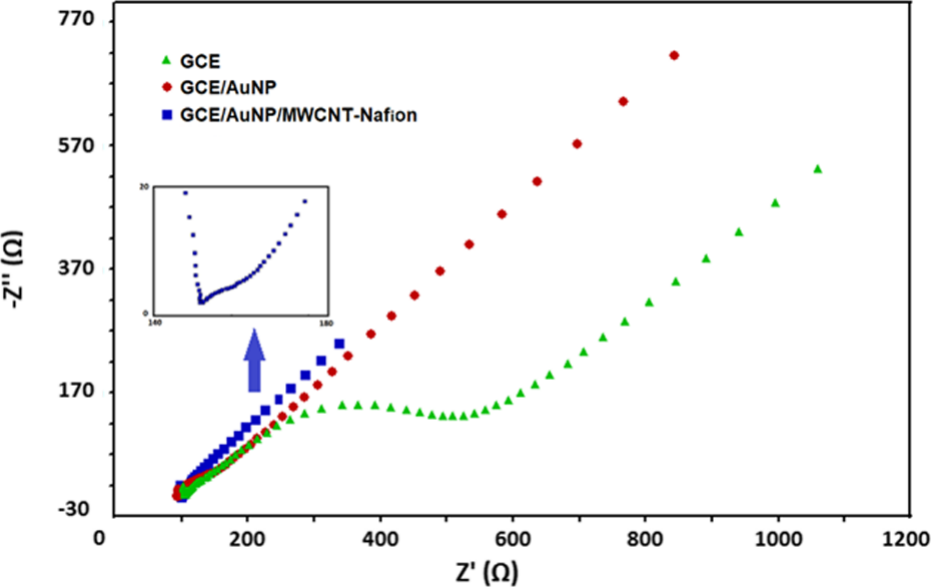
Nyquist plots of bare GCE, AuNP, and AuNP/MWCNT-Nafion-modified electrodes in a 0.1 M KCl solution containing 5 mM Fe(CN)_6_^3–/4–^ (frequency range: 10^6^–10^–1^ Hz and amplitude: 5 mV).

EIS data of bare GCE, AuNP, and AuNP/MWCNT-Nafion-modified GCEs were recorded in a 0.1 M KCl solution containing a 5 mM Fe(CN)_6_^3–/4–^ redox probe and are given in Figure 4 as a Nyquist plot. The bare GCE exhibited a semicircle on the electrode surface in the low-frequency region with significant resistance to the electron transfer process, whereas AuNP and AuNP/MWCNT-Nafion-modified electrodes exhibited a small semicircular region, indicating very low impedance of the nanoparticles. It was observed that the electron charge resistances (*R*_ct_) obtained after modification were less than those of the GCE (*R*_ct_ = 403.1 Ω). This value was found to be 50.1 Ω for AuNP modification, which was further reduced to 10.2 Ω by incorporating MCNT-Nafion (AuNP/MWCNT-Nafion) to the surface (from the diameter of the semicircle, which is also shown as the inset in Figure 4). As a result, it was observed that electron transfer rates increased and *R*_ct_ values decreased due to the high conductivity of AuNP and MWCNT when a plain GCE was modified with AuNP and MWCNT-Nafion to yield GCE/AuNP/MWCNT-Nafion. These results support the findings with the CV method, meaning that the nanomaterial combination increased the conductivity of the bare electrode and improved its electrochemical properties. Wu et al.^41^ have stated that AuNPs can provide a platform for DNA immobilization and increase electrical conductivity synergistically with MWCNTs. Indeed, our findings also confirmed that the nanomaterial combination may ameliorate the conductivity and electrochemical properties of the bare electrode.

**Figure 5:**
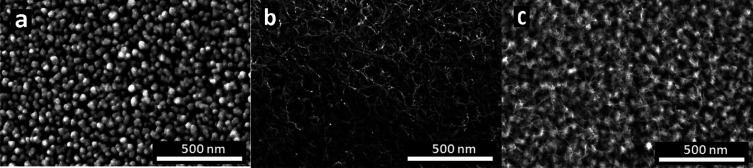
SEM images of modified electrodes: (a) GCE/AuNP, (b) GCE/AuNP/MWCNT-Nafion, and (c) GCE/AuNP/MWCNT-Nafion/DNA (magnification: 100,000×; AuNP sizes: 30–150 nm; and dsDNA: 250 mg/L immobilized).

After the modified surfaces were electrochemically compared, examination of surface topographies was made using SEM so as to observe the changes on electrode surfaces. For this purpose, SEM images of AuNP and AuNP/MWCNT-Nafion-modified electrodes were first taken without DNA immobilization (Figure 5a,b). Then, 250 mg/L dsDNA was immobilized (deposition) on the electrode surface modified with AuNP/MWCNT-Nafion, and the SEM image is shown in Figure 5c. When the surface topographies were examined, it was seen that there was a change after 30 cycles of AuNP (Figure 5a) and AuNP/MWCNT-Nafion (Figure 5b) modification. In the SEM images of the AuNP/MWCNT-Nafion-modified GCE, a clear and homogeneous distribution of MWCNTs in the polymeric mesh was seen. DNA immobilization gave rise to the formation of DNA clusters on the MWCNT, indicating successful operation.

### 3.4 Optimization of the Experimental Conditions

The parameters affecting the immobilization of DNA on the electrode surface (concentrations of DNA and supporting electrolyte solution, pH, and DPSV method parameters) and copper-catalyzed damage (Cu(II) concentration, interaction time, etc.) were investigated. First, the concentration of the dsDNA solution (125–1000 mg/L) to be used in the experiments was examined. The optimal concentration was determined as 250 mg/L because sensitivity decreased at higher concentrations (500, 750, and 1000 mg/L). This can be explained by the saturation of the DNA transported to the electrode surface under diffusion control. Then, the supporting electrolyte solution concentration (25–100 mM) and pH (4.8, 7.4, and 8.5) were optimized. The optimal pH (7.4) and supporting electrolyte concentration (75 mM) were determined. Finally, the parameters affecting the DPSV technique developed for the immobilization of DNA (deposition potential: −0.6 to 1.0 V, deposition time: 30–420 s, pulse size: 10–120 mV, and step size: 2, 5–15 mV) were examined. As a result, optimal deposition potential (0 V), deposition time (180 s), pulse size (75 mV), and step size (10 mV) values were determined.

The effects of the Cu(II) concentration (0.75–3.0 mM) and interaction time (5–40 min) on copper-catalyzed damage were investigated; the optimal Cu(II) concentration (1.5 mM) and interaction time (30 min) were determined for DNA damage on the modified GCE. It was originally intended to determine the AO effect that prevents the formation of DNA damage. Two types of applications were made to determine the difference between the addition of AO compounds to the system before and after Cu(II)-catalyzed damage formation, that is, whether they have protective or restorative effects. In the first approach, AO (RA, GA, and CT) was initially added to the DNA solution, and the occurrence/attenuation of Cu(II)-catalyzed damage was tested. In the second approach, Cu(II)-catalyzed DNA damage was induced first, and the effect of added AO compound to repair the damage was examined. In the first experiment, 0.05 mM AO was initially added to a 250 mg/L DNA solution, and then a 1.5 mM Cu(II) solution was added (30 min incubation). In the second experiment, a 1.5 mM Cu(II) solution was added to a 250 mg/L DNA solution (30 min incubation), and then 0.05 mM AO was added (30 min incubation). The findings, based on the oxidation peak current values of the adenine base, showed that the AO protective effect was significantly higher in the case involving AO addition before interaction with Cu(II) (Table 1).

**Table 1:** Investigation of the Protective and Repair Effects of AOs in the Interaction of DNA with Cu(II)

assays	–*I*_A_ (μA)^a^
DNA	47.1 ± 1.2
damaged DNA	4.6 ± 0.2
Protective Effect of AOs	
RA	17.0 ± 0.5
GA	29.2 ± 1.5
CT	33.2 ± 1.9
Repair Effect of AOs	
RA	15.8 ± 0.6
GA	25.5 ± 1.4
CT	28.4 ± 1.4

a Oxidation peak current values of the adenine base ± standard deviation.

**Figure 6:**
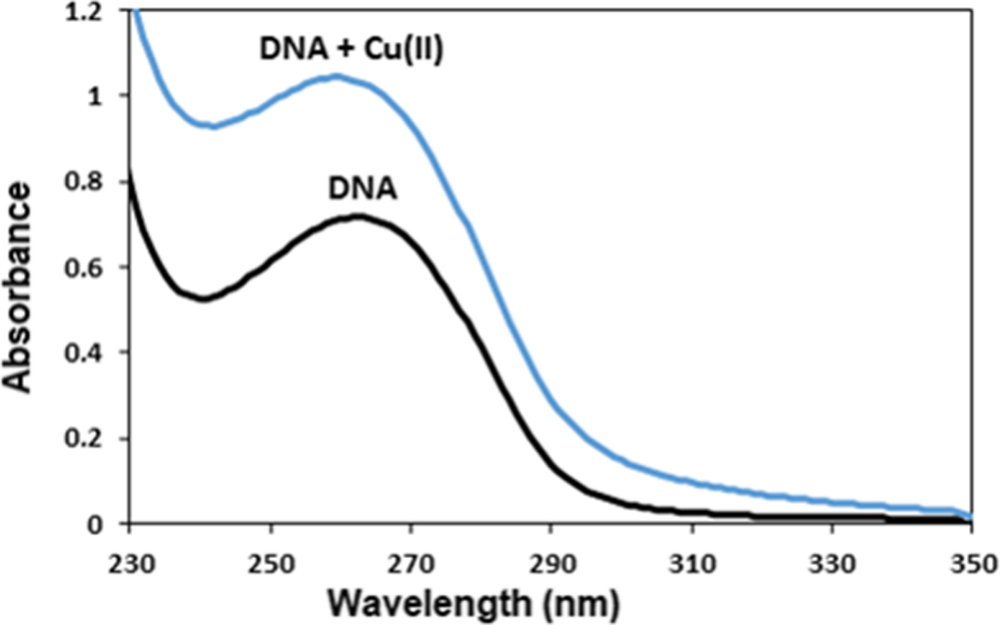
UV–vis spectra of the dsDNA solution before and after interaction with an aerated Cu(II) solution (10-fold diluted).

On the other hand, UV–vis region spectra of undamaged and damaged (Cu(II)-catalyzed) DNA solutions were presented as evidence of oxidative damage in DNA. Literature data state that 260 nm is the characteristic wavelength of DNA due to π → π* transition of DNA bases and that DNA double-strand breaks provide an increase in absorption at 260 nm, i.e., a hyperchromic effect that can be used to evaluate the level of DNA damage. In our study, an increase in absorbance values at 260 nm was observed in the spectrum obtained after the interaction of dsDNA with Cu(II) (Figure 6), confirming that the DNA was damaged and underwent fragmentation and degradation.^42^

### 3.5 Electrochemical Detection of DNA Damage in the Copper-Catalyzed System

Measurements were taken with the GCE/AuNP/MWCNT-Nafion electrode using standard solutions of G, A, and C bases at different concentrations. Thus, the oxidation potentials determined for each base in the DNA solution were verified with the standards. Calibration graphs were created between concentrations and oxidation peak currents for each standard. A linear range was determined as 12.5–125 mg/L for the G, A, and C standards {(I_A_(μA) = 6.12[A(mg/L)] + 1.22; *r* = 0.999), (I_G_(μA) = 2.24[G(mg/L)] + 9.01; *r* = 0.998), and (I_C_(μA) = 0.35[C(mg/L)] + 2.11; r = 0.997)}. Oxidative DNA damage was investigated by the Cu(II)-catalyzed damage method developed using the modified working electrode. In this method, it is thought that the radical mixtures (e.g., O_2_^•–^ and ^•^OH) are formed in the presence of Cu(II) and dissolved O_2_ (potentially via Cu(II)–DNA binding).^43^ Once Cu(II) was bound to DNA and reduced by biological reductants to Cu(I) in situ, Shao et al.^43^ demonstrated that the DNA–Cu(I) complex could react with H_2_O_2_ to form a DNA–Cu(I)–OOH complex, which was able to release ^•^OH to attack the neighboring DNA constituents in a site-specific manner.
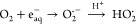

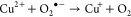

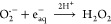

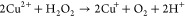

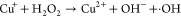


**Figure 7:**
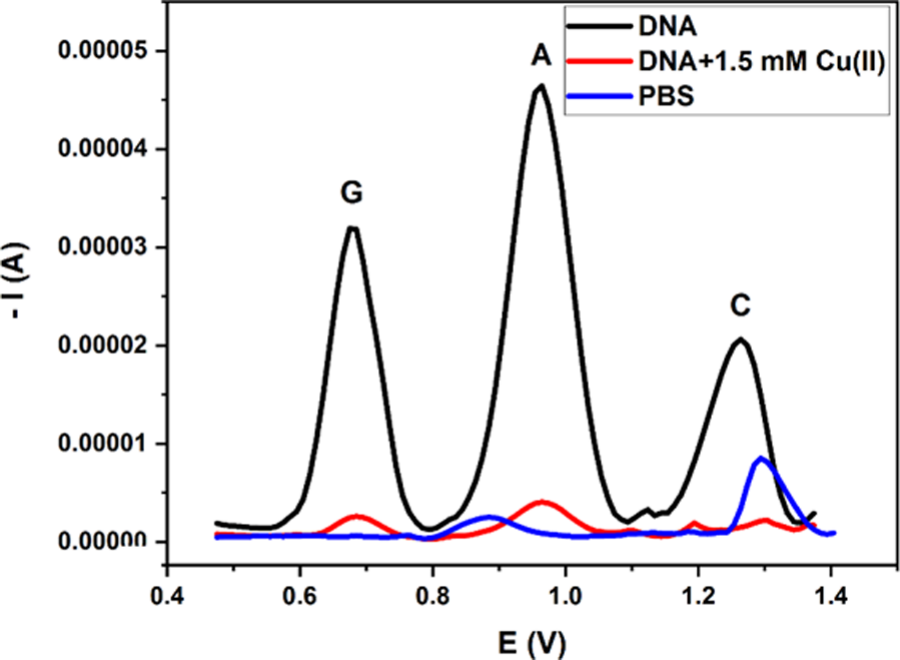
DPS voltammograms of PBS, dsDNA, and Cu(II) + dsDNA solutions obtained with the modified electrode (GCE/AuNP/MWCNT-Nafion) (scan range: 0.4–1.4 V; 75 mM pH 7.4 PBS).

Since these radicals cause damage to DNA, a decrease in the oxidation peaks of the G, A, and C bases was observed in electrochemical measurements. In other words, the oxidation current of DNA bases was significantly reduced in the presence of ROS compared to their natural electroactivity. Figure 7 shows DPS voltammograms of the DNA biosensor before and after interaction with the Cu(II) solution. On the other hand, the modified electrode could not detect any electroactive species in the blank PBS, i.e., in the absence of DNA.

The measured oxidation potentials and peak current values are given in Table 2, where the observed decrements in the peak current values of the G, A, and C bases in DNA indicated that Cu(II)-catalyzed radical formation occurred in the presence of dissolved oxygen and that these radicals damaged DNA. The measured peak currents varied proportionally with the amount of dsDNA left on the electrode and could therefore directly indicate dsDNA damage.^20^

**Table 2:** Oxidation Potentials and Peak Current Values Obtained for G, A, and C Bases in DNA (*n* = 6)

DNA bases	oxidation potential (V)	oxidation peak current (−*I*) (μA)^a^
Undamaged		
G	0.68	32.0 ± 1.2
A	0.96	46.0 ± 2.1
C	1.24	21.0 ± 0.9
Damaged		
G	0.70	3.0 ± 0.06
A	0.88	4.0 ± 0.08
C	1.24	n.d.

a Oxidation peak current values of DNA bases ± standard deviation.

The relative amount of oxidative damage to DNA caused by ROS formed in the solution medium was calculated with eq 1 using the measured peak current values. The results show that 90.9 and 91.2% of damage occurred in the G and A bases, respectively. In this approach, since a mixture containing various ROS was formed in the solution, close results of many repetitive experiments were evaluated. Our findings appear to be close to the damage (87.6%) reported by Barroso et al*.* arising from the Fenton reaction.^8^
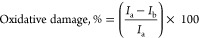
1where *I*_a_ is the oxidation peak current of bases in dsDNA and *I*_b_ is the oxidation peak current of bases in dsDNA after damage.

### 3.6 Determination of AO Ability in Preventing DNA Damage

**Figure 8:**
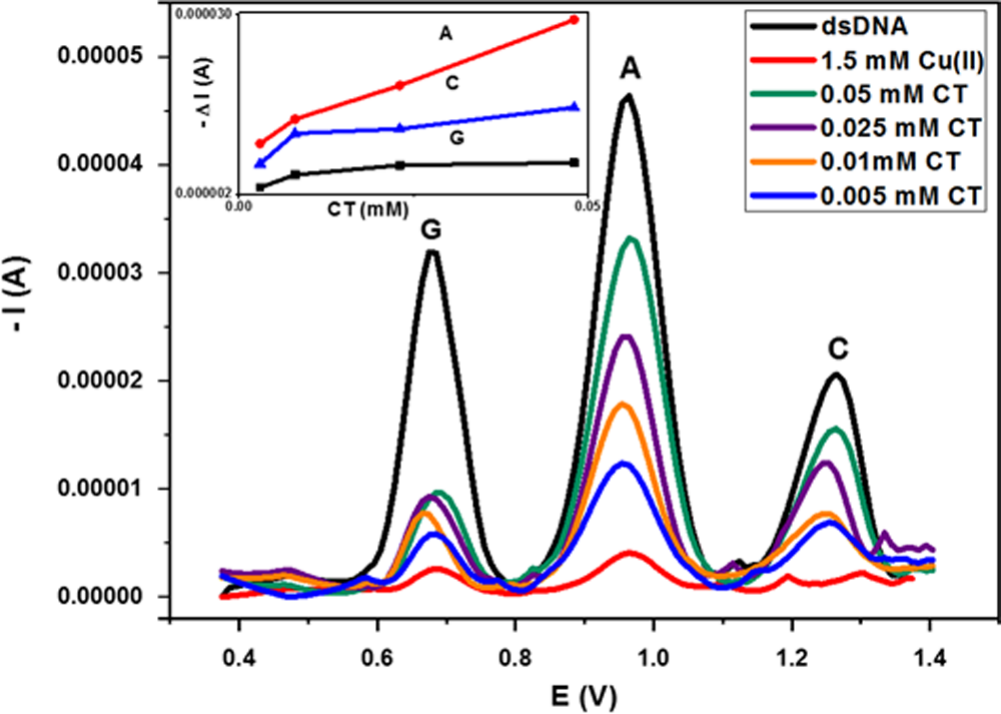
DPS voltammograms obtained with the modified electrode (GCE/AuNP/MWCNT-Nafion) (of 250 mg/L dsDNA; before interaction with a 1.5 mM Cu(II) solution, after 30 min interaction), in the presence of 0.005–0.05 mM CT (after 30 min interaction).

The AOs found in foodstuffs make an excellent natural source to counter and prevent the harmful effects of ROS. Many natural and synthetic AOs are known to exhibit effective protection against DNA damage.^44,45^ Indeed, when an AO is added to a solution containing reactive species, the current corresponding to the scavenging activity of these compounds is partially recovered. This protective effect is analytically defined as a special case of AOA. The protective (AO) effect determined here is due to AOs’ scavenging reactive species formed in the presence of Cu(II) and dissolved oxygen. In this study, oxidation peak currents of dsDNA that remained relatively intact in the presence of AOs were examined, and the activities of each AO to prevent oxidative DNA damage were determined. DPS voltammograms obtained using the modified electrode in the presence of different concentrations of CT standards are shown in Figure 8. In order to determine the protective effects of AO compounds, the voltammetric signal of base A was taken as a basis, and the concentration ranges enabling a linear increase in peak currents (i.e., AO ability) were determined.

The difference of oxidation peak currents (Δ*I* = *I*_c_ – *I*_b_) measured in the absence (*I*_b_) and presence (*I*_c_) of AO for each concentration was calculated, and the calibration equations of the AOs were obtained (Table 3).

**Table 3:** Calibration Equations, Correlation Coefficients, LOD and LOQ Values, and Working Concentration Ranges of the Protection of the A Base in dsDNA Depending on the Oxidative Damage Preventive Activities of Various AOs (*n* = 6)

AO	working ranges (μM)	calibration equations^a^	*r*	LOD^b^ (μM)	LOQ^c^ (μM)
QR	5–50	*y* = 0.421*x* + 11.14	0.995	1.0	3.0
CT	5–50	*y* = 0.419*x* + 8.38	0.999	1.0	3.0
RT	5–50	*y* = 0.387*x* + 10.46	0.994	1.0	3.0
GA	5–50	*y* = 0.314*x* + 9.95	0.995	1.0	4.0
ECG	10–100	*y* = 0.231*x* + 7.45	0.992	4.0	13.0
EC	5–50	*y* = 0.209*x* + 8.21	0.999	2.0	6.0
CA	10–100	*y* = 0.169*x* + 11.67	0.996	4.0	13.0
NAC	10–100	*y* = 0.166*x* + 10.87	0.998	5.0	17.0
AA	25–250	*y* = 0.064*x* + 11.63	0.999	4.0	14.0
GSH	10–250	*y* = 0.059*x* + 12.93	0.998	4.0	13.0
RA	10–250	*y* = 0.052*x* + 9.45	0.993	5.0	17.0

a *y* = – Δ*I* (μA) and *x* = μmol/L

b LOD = 3*s*_bl_/*m* (*m*: the slope of the calibration line and *s*_bl_: the standard deviation of the blank).

c LOQ = 10*s*_bl_/*m*.

**Figure 9:**
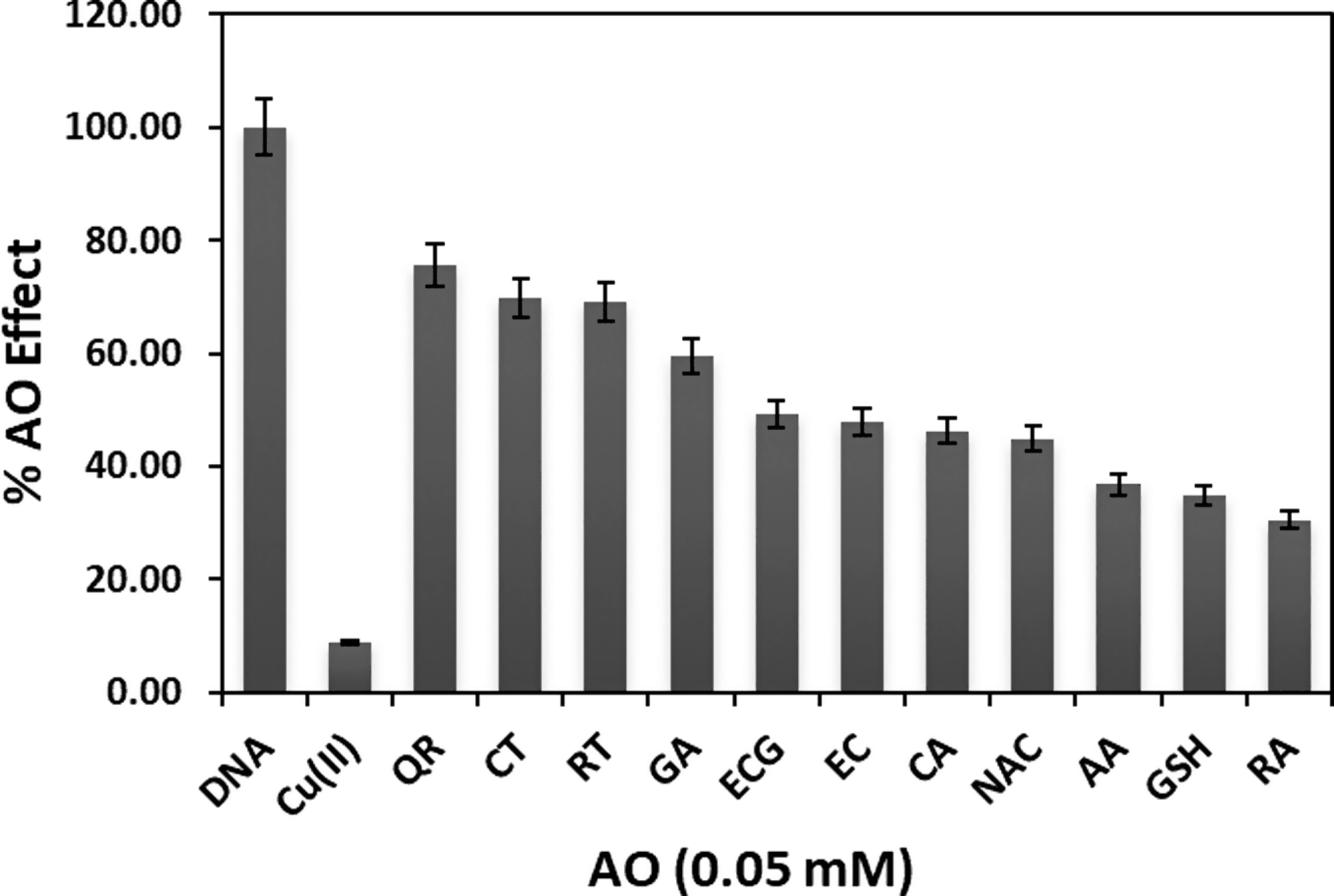
Comparison of ROS scavenging efficiencies (% AO effect) of various AO compounds (0.05 mM): QR—quercetin; CT—catechin; RT—rutin; GA—gallic acid; ECG—epigallocatechin; EC—epicatechin; CA—chlorogenic acid; NAC—*N*-acetyl cysteine; AA—ascorbic acid; GSH—glutathione; and RA—rosmarinic acid (*n* = 6).

Using the oxidation peak current values of the A base corresponding to the same concentration value (0.05 mM) of each AO compound, the AO effects were calculated (eq 2).^46^ In this equation, *I*_a_ denotes the electrocatalytic current (expected maximum value) measured before damage, and *I*_b_ and *I*_c_ denote the current intensities measured after DNA damage in the absence and presence of the AO compound, respectively. The results were compared in the form of a bar diagram, as shown in Figure 9.
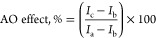
2

Experimental studies showed that AOs did not have the same effect on every DNA base. For example, a more linear protection was obtained for base A. Similarly, it was reported in the literature that the electrochemical quantification of oxidative alterations in DNA mostly relies on the detection of the oxidation peaks of purine bases.^36^ Among all compounds tested, QR, CT, and RT showed the highest AO effect (75.6, 69.7, and 68.9%, respectively) (Figure 9).

This is in accordance with previously reported structure–activity relationships that the 3-OH group of QR and RT strongly affects AO ability, possibly via their effects on planarity.^47^ In this case, QR and RT, which allow for a more planar and conjugated structure, appear to have stronger AOA. The flavonoid radical expected to emerge from the free 3-OH group of QR would have sufficient stability, resulting in stronger AOA compared to other AOs without free 3-OH. The lowest AO effect was found to be 34.7 and 30.5% for GSH and RA, respectively. The order of AOAs observed here is partially compatible with that of the cupric reducing antioxidant capacity (CUPRAC) method available in the literature,^48,49^ confirming that the modified electrode and the developed method gave successful results in the measurement of AO ability. The AO ability of RA was an exception in the efficacy order, as it ranked as a powerful AO in the CUPRAC method compared to its lower rank in the current method. This can be explained by the possible repulsive effect of the Nafion-modified electrode (having negatively charged sulphonate groups) on the negatively charged RA because its p*K*_a_ is 3.13, and at the measurement pH of 7.4, RA is monoanionic (conjugate base). It may be concluded that AOs have protective effects on DNA damage in the current method within certain concentration ranges.

### 3.7 Determination of AO Abilities of Herbal Teas on DNA Protection

Antioxidative abilities of some herbal teas (green tea, linden, apple tea, and echinacea tea) preventing oxidative damage were investigated with the prepared modified electrode. The extracts of the examined herbal teas were obtained by microwave extraction, which is a more effective technique than traditional extraction techniques. Significantly shortened extraction time, improved extraction efficiency, and usage of a lower amount of solvent are the advantages of this application.^50^ AOA was calculated in CT equivalents using the measured ΔI current values for real samples (0.05 mL) (eq 3). AOA values for green tea, linden, echinacea, and apple tea were found to be 80.3, 75.8, 70.9, and 62.1 μmol CT/g, respectively. Green tea contains bioactives comprising various constituents having AOA, such as polyphenols (CT, ECT, and EGCT) and vitamins.^51,52^ This content may explain the high AOA and DNA protective effect of green tea, in accordance with literature findings.
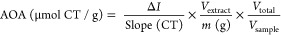
3

**Figure 10:**
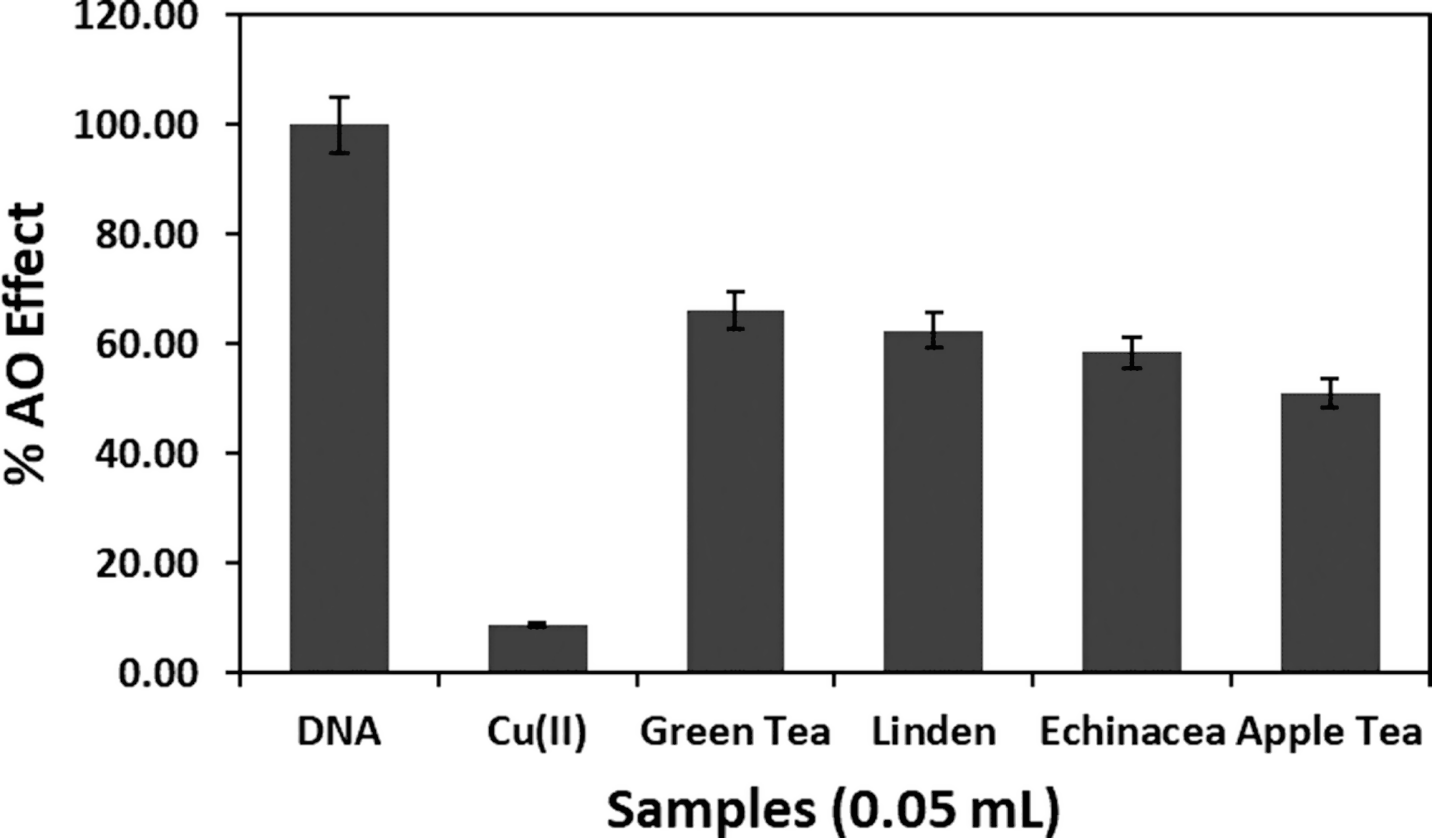
Comparison of the radical scavenging efficiencies (AO effect, %) obtained by the developed electrochemical method for herbal tea extracts (0.05 mL) (*n* = 6).

The relative AO abilities of green tea, linden, echinacea, and apple tea were found to be 66.2, 62.5, 58.4, and 51.1%, respectively (Figure 10). The results show that the developed method can be recommended to elucidate the DNA damage preventive AOA profile of food samples.

### 3.8 Comparison of the Developed Electrochemical Method with the HPLC-PDA Method

The performance of the developed electroanalytic method was compared against that of an HPLC method available in the literature.^29^

**Figure 11:**
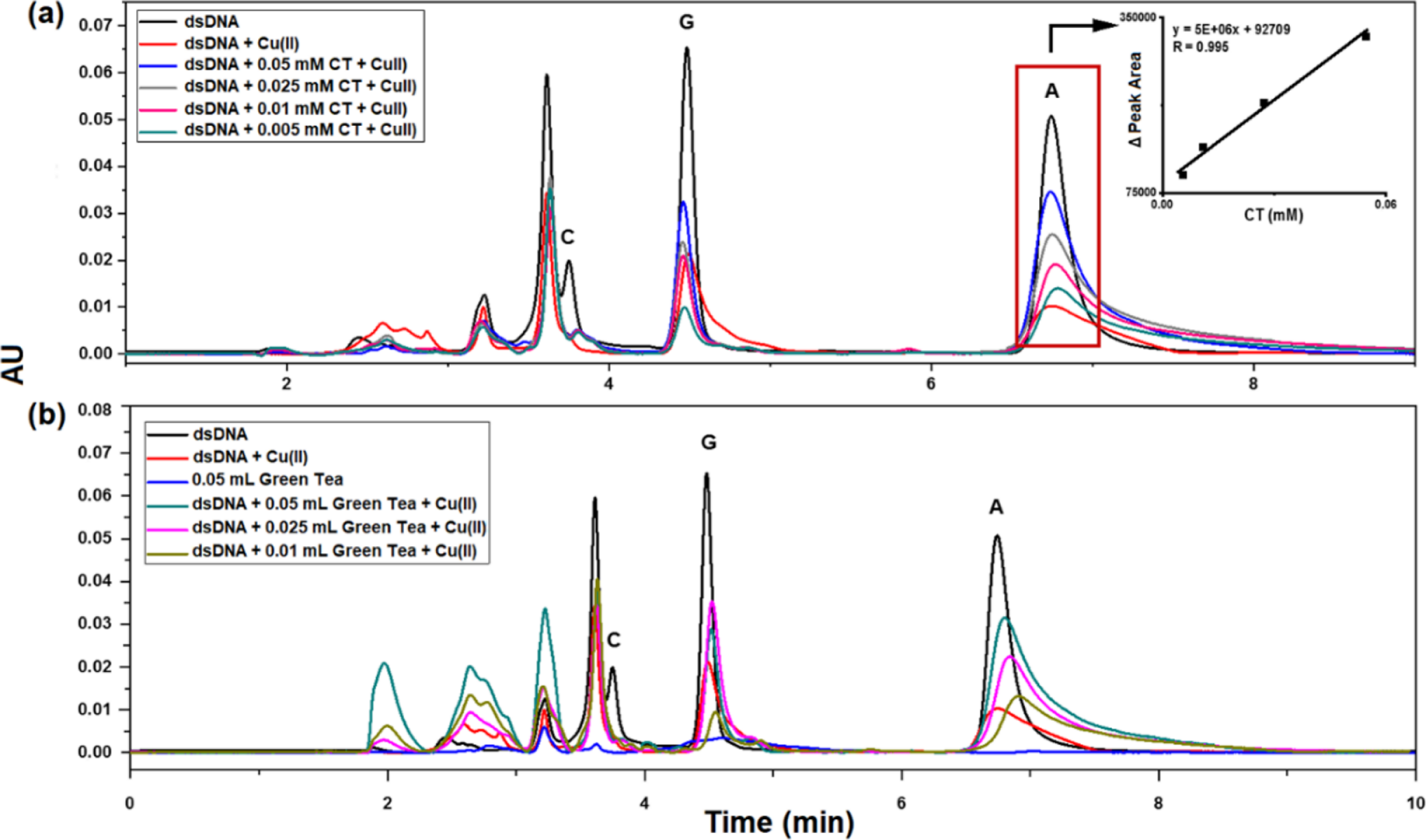
HPLC-PDA chromatograms [250 mg/L dsDNA, after interaction with 1.5 mM Cu(II), after interaction with 1.5 mM Cu(II) in the presence of CT (a) and green tea (b)] (λ = 254 nm; *n* = 3).

This study was performed by measuring the CT standard and the green tea sample simultaneously using both methods. Inspection of the DNA chromatograms revealed that there was effective damage to the DNA bases after 30 min of interaction with a 1.5 mM Cu(II) solution, similar to the observation made with the developed electroanalytic method (Figure 11a). CT (0.005–0.05 mM) and green tea (0.01–0.05 mL) were seen to have a particularly strong AO effect on the A base, as in the developed DPSV method (Figure 11b). The AO effect values (eq 4) for green tea (0.05 mL) and CT (0.05 mM) were calculated as 61.99 and 62.22%, respectively.
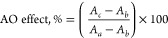
4where *A*_a_: peak area of base A in dsDNA (undamaged); *A*_b_: peak area of base A in dsDNA after damage in the absence of AO; and *A*_c_: peak area of base A in dsDNA after damage in the presence of AO.

The results obtained with the HPLC-PDA and DPSV methods were compared statistically (*n* = 5) to observe compatible findings between these methods. The *F*-test between the DPSV and HPLC-PDA methods revealed that there was no significant difference at the 95% confidence level in terms of precision (CT standard: *P* = 0.05, *F*_experimental_ = 0.0901, *F*_critical(table)_ = 5.5914, and *F*_experimental_ < *F*_critical(table)_; green tea: *P* = 0.05, *F*_experimental_ = 0.4882, *F*_critical(table)_ = 5.5914, and *F*_experimental_ < *F*_critical(table)_). Moreover, the developed electroanalytical method was compared with the reference method in terms of repeatability (relative standard deviation, RSD) and recovery (REC). Comparison of intraday and interday RSD (%) and REC (%) values is given in Table 4. The results show that the two methods are compatible and reproducible.

**Table 4:** Reproducibility (Intraday and Interday) and Recovery Values Obtained by Comparing the Developed DPSV and HPLC-PDA Reference Methods (*n* = 6)

method	REC (%)	RSD (%) (intraday)	RSD (%) (interday)
DPSV			
G	98.9	4.6	3.5
A	98.3	3.4	1.9
C	97.3	4.2	4.8
HPLC-PDA			
G	99.2	1.7	3.5
A	101.1	1.1	2.4
C	102.5	4.0	5.8

### 3.9 Interference Effects on the DPSV Method

In the determination of 250 mg/L dsDNA using the DPSV method employing the GCE/AuNP/MWCNT-Nafion electrode, possible interference effects on the determination were investigated based on the A oxidation peak current value. The amount of FBS that did not show any interference effect was determined as 0.01 mL of a 1:1 (v/v) dilute solution. The error values (%) calculated for this matrix (before interaction with Cu(II), after the interaction, and in the presence of GA) are, respectively, (−2.4), (+3.9), and (−0.8). The calculated error % values of studied compounds are (+2.6), (+3.2), and (+1.2) for DA; (−3.6), (+9.2), and (+8.9) for UA; (−1.2), (+1.0), and (+1.3) for BSA; and (−1.1), (+3.1), and (−1.4) for glucose.

### 3.10 Comparison of the Electrochemical Assay Results with Those Available in the Literature

Voltammetric methods are suitable and widely preferred for studying DNA oxidation. However, when similar studies in the literature are examined, it is seen that most of them focus only on the oxidation of purine derivative compounds. These are applications using different electrodes and voltammetric methods. In some of them, only DNA analysis was performed, and the protective AO effect was not evaluated. Comparison of these applications with the developed method is shown in Table 5.

**Table 5:** Comparison of the Current DPSV Method with Similar Electrochemical Applications Available in the Literature

electrode	damaging reagent	method	target	AO	AO effect (%)	references
DNA/SPE	Fe^2+^:EDTA:H_2_O_2_	SWV	G	TR and plant samples	38–75^a^	(20)
chitosan/DNA/CFE	H_2_O_2_:Cu(phen)_2_(ClO_4_)_2_ XA/XOD	SWV	G and A	apple and orange juice	60–90	(21)
DNA/PGE	Fe^2+^:EDTA:H_2_O_2_	DPV	G	NAC, GSH and HCYS		(22)
DNA/GCE	dopamine/Fe^3+^	DPV		AA and RT		(53)
DNA/CPE	Fe^2+^:EDTA:H_2_O_2_	DPV	dA_21_	AA, GA, TR, CFA, RES and beverages	19–59	(8)
DNA/CPE	XOD/xanthine	CV	dA_21_	AA, GA, TR, CFA, RES and beverages	33–63	(44)
DNA/PAMAM-Au-Pd/CHIT/GCE	Fe^2+^:EDTA:H_2_O_2_	SWV	G	sericin and TR	70–89	(46)
CB/P[5]A/poly-NR film/DNA/GCE	Cu(II)/H_2_O_2_	EQCM	DNA	AA		(28)
DNA/AuNPs/SPE	Cu(II)/H_2_O_2_	DPV	DNA	*Acanthophora* red macroalgae, AA		(7)
SH-DNA/AuNPs/SPGE	Cu(II)/H_2_O_2_/AA	CV, EIS	DNA	deferoxamine (DFO)		(54)
MWCNT-Nafion/AuNP/GCE	Cu(II) + dissolved O_2_	DPSV	G, A, and C	QR, CT, RT, ECG, CA, EC, GA, AA, NAC, GSH, RA	30–75	this work

a This value was calculated by us according to the results in the literature.

## 4 Conclusions

The developed electroanalytical method can be described as a successful approach that gives fast and reliable results, enabling the detection of DNA bases at high current values and measuring both the Cu(II)-catalyzed oxidative damage on DNA and its protection by AOs. Most hydrogen atom transfer- and electron transfer-based AO assays are criticized because of using artificial probes for measuring the defense against reactive species, but this assay uses a biologically relevant probe such as DNA. Most of the studies in the literature conducted with voltammetric methods focus only on the oxidation and damage of purine-derived compounds. With this work, these voltammetric studies for DNA analysis have been expanded to include three bases (A, G, and C). In addition, a comprehensive method has been proposed that can measure the AO ability of various compounds reflected in the prevention of oxidative DNA damage induced by Cu(II). The results of this study may help to better understand the biotoxicity mechanism of copper, an essential trace element in the center of certain metalloenzymes and yet an inducer of oxidative damage to DNA, proteins, and lipids and therefore a potential contributor to disease pathology. For determining the damage on DNA, a novel modified electrode (GCE/AuNP/MWCNT-Nafion) was prepared using the CV method, and its characterization was performed with CV, EIS, and SEM analyses. The immobilization and electrochemical behavior of DNA were examined by the DPSV method. AO compounds were tested as ROS scavengers with efficiencies ranging from 30.5 to 75.6%, and QR showed the highest protective role in accordance with literature findings. Among the plant extracts whose AO effect was examined, green tea, which is known to contain polyphenols (CT, ECT, and EGCT) and vitamins, was found to have the highest protective effect (80.3 μmol CT/g).
